# Locus coeruleus TDP-43 pathology in a community-based cohort: Clinical and pathological correlates

**DOI:** 10.1093/jnen/nlag035

**Published:** 2026-04-20

**Authors:** Allison M Neltner, Ryan K Shahidehpour, Megan E Hall, Xiaotong Ning, Shuling Fister, Hannah Kang, Sonya Anderson, Gregory A Jicha, Tiffany L Lee, Erin L Abner, David W Fardo, Peter T Nelson

**Affiliations:** Sanders-Brown Center on Aging, University of Kentucky, Lexington, KY, United States; Sanders-Brown Center on Aging, University of Kentucky, Lexington, KY, United States; Sanders-Brown Center on Aging, University of Kentucky, Lexington, KY, United States; Department of Biostatistics, University of Kentucky, Lexington, KY, United States; Sanders-Brown Center on Aging, University of Kentucky, Lexington, KY, United States; Department of Epidemiology and Environmental Health, University of Kentucky, Lexington, KY, United States; Sanders-Brown Center on Aging, University of Kentucky, Lexington, KY, United States; Sanders-Brown Center on Aging, University of Kentucky, Lexington, KY, United States; Sanders-Brown Center on Aging, University of Kentucky, Lexington, KY, United States; Sanders-Brown Center on Aging, University of Kentucky, Lexington, KY, United States; Department of Neurology, University of Kentucky, Lexington, KY, United States; Sanders-Brown Center on Aging, University of Kentucky, Lexington, KY, United States; Sanders-Brown Center on Aging, University of Kentucky, Lexington, KY, United States; Department of Epidemiology and Environmental Health, University of Kentucky, Lexington, KY, United States; Sanders-Brown Center on Aging, University of Kentucky, Lexington, KY, United States; Department of Biostatistics, University of Kentucky, Lexington, KY, United States; Sanders-Brown Center on Aging, University of Kentucky, Lexington, KY, United States; Department of Pathology, University of Kentucky, Lexington, KY, United States

**Keywords:** aging, anxiety, digital pathology, HALO, locus coeruleus, NPI-Q, TDP-43

## Abstract

The locus coeruleus (LC) is the source of norepinephrinergic innervation in the human brain. Locus coeruleus pathology has been linked to clinical conditions such as cognitive impairment and behavioral and psychiatric symptoms of dementia (BPSD). However, phosphorylated TDP-43 (pTDP-43) pathology in the LC has been understudied, particularly in the contexts of aging and limbic predominant age-related TDP-43 encephalopathy neuropathologic change (LATE-NC). Here, a convenience sample (*n* = 134) of autopsied participants from the University of Kentucky Alzheimer’s Disease Research Center community-based cohort was analyzed for LC pTDP-43 and phosphorylated tau (pTau) pathologies. Locus coeruleus pTDP-43 pathology was found in 28/134 (20.9%) brains and was generally sparse when present. Locus coeruleus pTDP-43 lesions typically appeared as round granular structures that measured ∼3-12 µm in diameter. Locus coeruleus pTDP-43 pathology was increased with aging and was imperfectly correlated with LATE-NC staging; it showed no correlation with cortical pTau pathology in ADNC or PART. In terms of clinical-pathological correlations, LC pTDP-43 pathology was associated with depressive symptoms but not with global cognition or with other BPSDs. Locus coeruleus pTau pathology was positively correlated with cortical pTau pathologic severity and with LATE-NC stages. In conclusion, LC pTDP-43 pathology is a common feature of brain aging, associated with LATE-NC.

## Introduction

The present study focused on locus coeruleus (LC) pathology in aging humans. The LC is a nucleus located within the pontine tegmentum. Although the LC measures only <15 mm in rostro-caudal extent and <3 mm in other dimensions, its neurons generate widespread axonal norepinephrinergic input for the brain and spinal cord.^1^ This small brainstem nucleus plays key roles in arousal, attention, memory, sleep-wake regulation, and other functions.^2–6^

Pathology in the LC has been implicated in neuropsychiatric disorders including depression.^2^^,^^5^^,^^7–9^ The LC also is vulnerable to cell loss and proteinopathy during the course of neurodegenerative diseases. More specifically, LC pathologies have been described in the contexts of Alzheimer disease neuropathologic change (ADNC), dementia with Lewy bodies, Parkinson disease, and frontotemporal lobar degeneration (FTLD).^10–17^ The LC appears to be the first site of phosphorylated tau protein pathology in most aging brains, including those with ADNC and in primary age-related tauopathy (PART).^18^ The severity of LC cell loss is associated with the cerebral pTau pathologic burden.^18–22^ In terms of clinical-pathological correlation, LC neurodegeneration has been linked to cognitive impairment, both in vivo (ie, according to clinical imaging),^23^^,^^24^ and in autopsy series.^21^^,^^25^^,^^26^ In addition to global cognition and memory symptoms, LC degeneration may contribute to the behavioral and psychiatric symptoms of dementia (BPSD).^27^^,^^28^

In spite of its widely recognized significance in neurodegenerative diseases, the LC remains understudied in the context of TAR-DNA binding protein of 43 kDa (TDP-43) pathology and limbic-predominant age-related TDP-43 encephalopathy neuropathologic change (LATE-NC). LATE-NC affects approximately one-third of autopsied individuals over the age of 85 years and is associated with a distribution of phosphorylated TDP-43 protein (pTDP-43) pathology that is usually restricted to the medial temporal lobe (MTL).^29^^,^^30^ Approximately 15% of those with LATE-NC show pTDP-43 proteinopathy outside the MTL, including in both neocortical and subcortical regions as first demonstrated by Josephs et al.^30–32^ However, the full extent of aging-related pTDP-43 pathology, and particularly the involvement of the LC, is incompletely understood.

There also remains some uncertainty about how the various prevalent subtypes of dementia-associated brain pathologies coexist and interact with one another. To date, the primary source of insights into these phenomena has been large autopsy series that have enabled clinical-pathology and pathology-pathology correlations with an epidemiologic scope. Whereas LATE-NC is robustly associated with the severity of cognitive impairment independent of other pathologies,^33^ LATE-NC also tends to be comorbid with various other conditions such as hippocampal sclerosis of aging (HS-Aging), brain arteriolosclerosis (B-ASC), and ADNC.^34^ Autopsy cohort data also indicate that pTDP-43 and pTau proteinopathies have synergistic influences and/or are both downstream of common risk factors.^35–38^ Additionally, in cases with comorbid pTDP-43 and pTau proteinopathies, cognitive decline tends to occur at an accelerated pace in comparison to cases only affected by one of those pathologies.^33^^,^^36^

Although recent studies have advanced our understanding of pTDP-43 pathology in aging brains, a focused investigation of LC pTDP-43 pathology in a community-based autopsy cohort has yet to be published. The current study aims to help bridge this knowledge gap and to evaluate the associations between LC pTDP-43 pathology and other cortically defined pathological features (eg, LATE-NC, ADNC) as well as with clinical phenomena (eg, global cognition, BPSD). To characterize LC pTDP-43 pathology and to test these associations, we evaluated a convenience sample of autopsied participants from the University of Kentucky Alzheimer’s Disease Research Center (UK-ADRC) cohort.

## Methods

### Study participants

The study sample was made up of research volunteers who came to autopsy as part of the UK-ADRC community-based cohort, which has previously been described in detail.^39^ Briefly, individuals were recruited after the age of 60 years and were followed through yearly longitudinal appointments until death when their brains were collected during autopsy.^39^^,^^40^ Informed consent was obtained from UK-ADRC research participants (and/or their legally authorized representatives) as approved of by the University of Kentucky Institutional Review Board. Certain exclusion criteria were applied at the time of initial recruitment, including parkinsonism, active substance use disorder, and severe neuropsychiatric disorder (eg, bipolar disorder or schizophrenia), and participants were followed thereafter, often over several decades, until death with eventual autopsy. Many participants became symptomatic (eg, with dementia, parkinsonism, BPSD, etc.) while on the study. Rare pathologic conditions such as prion disease, FTLD (including progressive supranuclear palsy, corticobasal degeneration), and multiple system atrophy were excluded from this study. All participants who came to autopsy from 2022 to 2024 and fulfilled inclusion criteria were included. To further enrich for high LATE-NC stage and PART, individuals with autopsy-confirmed LATE-NC stage 3 (individuals with pTDP-43 pathology in the middle frontal gyrus [MFG] but lacking FTD/FTLD) and participants with Consortium to Establish a Registry for Alzheimer’s Disease (CERAD) neuritic plaque scores of “no neuritic plaques,” who died after 2015 were also preferentially included in the convenience sample.

### Clinical diagnoses and parameters

The Neuropsychiatric Inventory Questionnaire (NPI-Q; UDS Form B5), part of the National Alzheimer’s Coordinating Center Uniform Data Set,^41–43^ was used to identify the presence or absence of 10 subtypes of BPSD (Table S1) in the 30 days prior to the study visit, as rated by a study partner. Behavioral and psychiatric symptoms of dementia symptoms from the NPI-Q included: agitation, anxiety, apathy, appetite problems, delusions, depression, disinhibition, hallucinations, and irritability. The NPI-Q was completed for all participants without regard to cognitive diagnosis. For the current study, each BPSD was considered present if the NPI-Q indicator was marked as present, regardless of the severity rating. The Clinical Dementia Rating (UDS Form B4)^44^^,^^45^ and Mini Mental State Exam (MMSE)^46^ were administered at each UDS visit as standardized measures of global cognitive status.^43^ Syndromic cognitive diagnoses (normal, mild cognitive impairment [MCI], impaired but not MCI, and dementia) were assigned annually at each UDS visit on consensus diagnosis of the UK-ADRC Clinical Core. All clinical data for the current study were drawn from the last UDS visit prior to death.

### Pathological diagnoses and readouts

Neuropathological protocols at the UK-ADRC were conducted as previously described.^47^^,^^48^ Briefly, after autopsy, tissue was fixed in 10% buffered formalin for approximately a month. Following fixation, rostral pons sections were cut at a level ∼1 cm from the pontine-midbrain junction. Tissue was embedded in paraffin and sections were cut at 8-μm thickness. Prior to further processing, tissue was baked onto glass slides overnight. Slides were deparaffinized in xylene and rehydrated in decreasing concentrations of ethanol. Heat-induced antigen retrieval was performed using a BioGenex EZ-Retriever System v.3 in a citric buffer (pH 6, DAKO TRS Lo #GV805). Immunohistochemical (IHC) assessments of Aβ (Nab228, a gift from Dr Edward Lee), pTau (PHF-1, a gift from Dr Peter Davies), pTDP-43 (1D3; BioLegend, Inc., Cat#829901), a second pTDP-43 (Proteintech Cat#80007-1-RR), nonphosphorylated TDP-43 (Proteintech Cat#80001-1-RR), GFAP (Agilent Tech, Cat#Z0334), and α-Synuclein (KM51; Leica Biosystems) were performed as previously described.^48–50^ Briefly, slides were blocked in 5% milk before the primary antibody was added. Sections were incubated with the primary antibody overnight at 4 °C prior to the addition of a biotinylated secondary antibody. Signal amplification was conducted using ABC amplification (Vectastain Elite ABC kit Peroxidase, Vector Laboratories #PK-6100) followed by visualization with 3,3-diaminobenzidine (DAKO #K3468) or NovaRED reagent (Vector Laboratories #SK-4805) per manufacturers’ instructions. Sections were counterstained using hematoxylin before being dehydrated, cleared, and mounted.

Where possible, assessments of the presence and severity of neuropathologies were diagnosed in accordance with consensus guideline recommendations. As suggested by Montine et al.,^48^ ADNC was gauged using 3 metrics: Braak neurofibrillary tangle (NFT) staging,^52^ Thal Aβ phases,^51^ and CERAD neuritic amyloid plaque scores.^53^ LATE-NC stages were assessed according to the distribution of pTDP-43 pathology as recommended in consensus guidelines.^29^ For this study, PART was defined as Thal Aβ phase 0 or 1 with Braak NFT stages ≤IV. Vascular pathologies, such as B-ASC severities and the numbers of grossly detected infarcts, were also recorded as previously described.^43^^,^^54^^,^^55^

### Analysis of LC pTDP-43 and pTau pathologies

The presence of pTDP-43 pathology was determined using a quantitative manual scoring method performed by a neuropathologist (P.T.N.) and independently reviewed by a second rater (A.M.N.). The manual counting approach was selected because automated quantification (as described below, for pTau quantification) was problematic in some cases due to the relative sparsity and subtle pTDP-43 pathology in the LC, as well as the brown pigment (neuromelanin, which was sometimes extracellular due to neurodegenerative processes) in that region. The LC pTDP-43 pathology was hence counted by eye and every lesion was parsed as 1 of 2 histomorphologic subcategories, that is, round granules (RGs) or other. The “other” category included a variety of subtypes of pTDP-43-immunoreactive pathology, including dystrophic neurites (DN), and skein-/neuronal cytoplasmic inclusion (NCI)-like structures that have been previously described.^56^ In cases where individuals were initially diagnosed as LATE-NC stage 0, but exhibited pTDP-43 pathology in the LC, stained sections in regions of the MTL (hippocampus and amygdala) were reexamined to ensure the LATE-NC staging accurately reflected the lack of pathology in these regions, and then serial sections of the LC were restained to confirm the presence of LC pTDP-43 pathology.

For pTau-immunostained slides, whole slide images (WSIs) were generated at 40× magnification using a Leica/Aperio AT2 Scanner. Due to the small size of the LC compared to the rest of the pons, particular care was taken to ensure that the LC was in focus, as this technical factor could impact digital analysis of WSIs. Given the presence of other pTau+ nuclei (such as the dorsal raphe), it was crucial to ensure that pTau+ NFT counts only originated from the LC. This region of interest (ROI), the LC, was hand circled according to anatomical references.^57^ A HALO AI DenseNet-based classifier was developed to specifically distinguish neuromelanin from pTau-immunoreactive NFTs in the LC. Regions of interest were subsequently analyzed using this classifier and NFT density was calculated as the number of identified objects divided by ROI area (square millimeter). Neurofibrillary tangle density in the cerebral cortex was derived from established Aperio image analysis methods as previously described.^58^^,^^59^ Neocortical NFT density was an average of the computer-generated NFT densities in 4 neocortical regions: MFG (Brodmann area [BA] 9), superior and middle temporal gyri (BA 21/22), inferior parietal lobule (BA 40), and occipital striate cortex (BA 17/18). MTL NFT density was calculated in the same way but using instead the NFT densities derived from 4 MTL regions: entorhinal cortex, amygdala, cornu ammonis 1 of the hippocampal formation, and subiculum (Table S2).

### Immunofluorescence staining

A subset of high LC pTDP-43 pathology cases was selected for immunofluorescence (IF) labeling for pTDP43 antibody (antibody raised in rat) and DNA via 4′,6-diamidino-2-phenylindole (DAPI), with or without costaining for GFAP (raised in rabbit; see above). Immunofluorescence labeling was performed on at least 2 paraffin-embedded serial sections of the pons, that is, one with and one without primary antibodies as controls for each case. Slides were first deparaffinized in xylene and then rehydrated through a graded series of ethanol (100%, 95%, and 80%) to distilled water. Antigen retrieval was performed in a homemade pH 9 buffer at 95 °C for 6 min, followed by a 10-min cooldown at room temperature. Slides were treated in formic acid for 3 min. Next, the slides were thoroughly rinsed in running distilled water for 10 min and equilibrated in 1× TBS for 5 min on a shaker. For blocking, sections were incubated for 1 h at room temperature in 1× TBS containing 5% goat serum. Primary rat antibody pTDP43 was diluted in buffer containing 5% goat serum and applied to pap-pen–circled tissue areas. One serial section per case was incubated with primary antibodies overnight at 4 °C; the other serial section functioned as a negative control and was incubated without primary antibodies. The following day, slides were washed twice in 1× TBS for 5 min each. Secondary antibody goat anti-rat IgG (Invitrogen A11007, Alexa Fluor 594) and/or goat anti-rabbit IgG (Invitrogen A11008, Alexa Fluor 48) were applied for 1 h at room temperature in the dark, followed by 2 additional 5-min washes in 1× TBS. To reduce lipofuscin autofluorescence, sections were incubated in 1× TrueBlack (ThermoFisher, 20× stock; 50 µL stock diluted in 1 mL 70% ethanol) for 10 min and rinsed thoroughly with 1× TBS until all residual dye was removed. Slides were then mounted using DAPI containing ProLong Gold Antifade Reagent (Invitrogen, P36935) and were cover-slipped. Immunofluorescence slides were visualized with a Nikon Eclipse E800 microscope and imaged with an attached Nikon DS Ri2 camera.

### Data analyses

Statistical analyses were performed using R, version 4.4.1.^60^ GraphPad Prism (v9) and R (v4.4.1) were used to generate graphs and heatmaps. Descriptive statistics, such as frequency, percentage, and mean with SD, were used to summarize the sample characteristics. Association between LC NFT density and BPSD variables were analyzed via Wilcoxon rank sum tests. Associations between LC NFT density with LATE-NC and Braak NFT stages were analyzed via Kruskal-Wallis tests.

Since >70% of the study sample had a zero count for LC pTDP-43 pathology, we chose to fit a hurdle model from the pscl package in R to quantify the association between LC pTCP-43 pathology and relevant clinical and pathological variables.^61^^,^^62^ The hurdle model consisted of 2 components: (1) a logistic regression (logit) model for the presence or absence of LC pTCP-43 pathology and (2) a truncated negative binomial (count) model for the total count of LC pTCP-43 pathologic lesions, if LC pTCP-43 pathology was present. The model includes covariates for age at death, sex, LATE-NC stage >1, and Braak NFT stage >IV. Hurdle models were fit for each BPSD variable (agitation, anxiety, apathy, delusions, disinhibition, elation, hallucinations, and sleep disorders), adjusting for the same covariates. For the logit portion of the hurdle models, odds ratios and 95% CIs are reported, and for the count portion, prevalence ratios and 95% CIs are reported.

## Results

### Workflow and study participants

**Figure 1:**
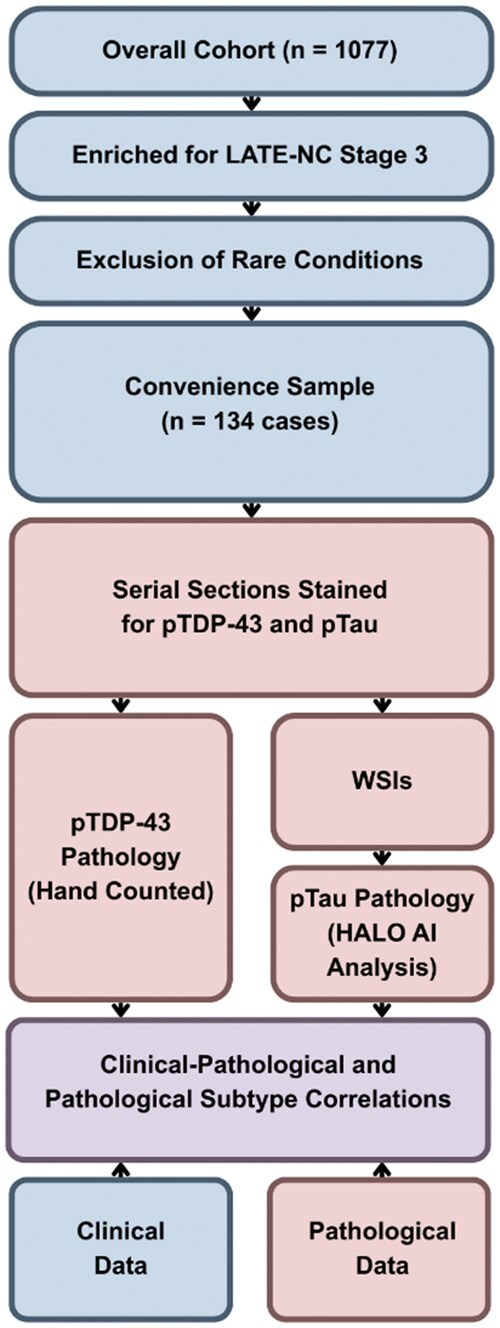
Workflow and study methodology. A convenience sample (*n* = 134) of participants was included from the University of Kentucky’s Alzheimer’s Disease Research Center (UK-ADRC) autopsy cohort. Sections of paraffin-embedded pons were stained using phosphorylated tau (pTau)- and phosphorylated TDP-43 (pTDP-43)-specific antibodies to determine the pathologic burden of each misfolded protein in the LC. Locus coeruleus pTDP-43 pathology was counted by eye under light microscopy. Locus coeruleus pTau-immunostained slides were converted to whole slide images (WSIs), which were analyzed using a HALO AI DenseNet classifier. These data were then compared to preexisting UK-ADRC data related to clinical (blue) and pathological (red) features.

The study workflow is described in Figure 1. Three sets of individuals from the UK-ADRC community-based autopsy cohort were sampled for the study, each fulfilling the following overall inclusion criteria (*n* = 145): (1) individuals who died and came to autopsy between 2022 and 2024 (*n* = 61), (2) those with a CERAD neuritic amyloid plaque score of “none” who died between 2015 and 2021 (to enrich for cognitively normal, PART, and *APOE* ε4- cases, *n* = 73), and (3) additional LATE-NC stage 3 individuals (*n* = 11) because our a priori hypothesis was that LATE-NC stage 3 cases would have more LC pTDP-43 pathology. In some participants (*n* = 10), an insufficient amount of LC tissue was available for review of the pons section; they were excluded from the study. One LATE-NC stage 3 individual had insufficient metadata for inclusion. Thus, the final study sample included *n* = 134 participants, of which *n* = 124 had replete BPSD data. Tables 1 and 2 display the demographics, clinical, and pathological characteristics of the included individuals. The participants and their various metrics are described stratified by cohort subsets in Table S3. The average age at death overall was 86.1 ± 7.9 (SD) years, and 51.5% (*n* = 69) of the included participants were female. Included participants spanned a range of clinical states at final clinic visit: normal (29.9%); MCI (23.1%); dementia (44.0%); and, impaired but not MCI (3.0%). Participants were followed with yearly longitudinal visits for 10.1 ± 6.7 years on average, with an average interval of 1.7 ± 1.7 years between their final study visit and death (Table 1).

**Table 1:** Selected demographic, clinical, and genetic information about included participants.

**Characteristic**	***n***	**Overall**
**Age at death^a^**	134	86.1 (7.9)
**Years on study^a^**	134	10.1 (6.7)
**Years between last clinic visit and autopsy^a^**	134	1.7 (1.7)
**Sex^b^**	134	–
** Male**		65 (48.5%)
** Female**		69 (51.5%)
**Diagnosis at final clinic visit^b^**	134	–
** Normal**		40 (29.9%)
** Impaired/Other**		4 (3.0%)
** MCI**		31 (23.1%)
** Dementia**		59 (44.0%)
**APOE-e4^b^**	126	–
** Carrier**		41 (32.5%)
** Noncarrier**		85 (67.5%)

Abbreviation: MCI, mild cognitive impairment.

a Mean (SD).

b *n* (%).

**Table 2:** Neuropathologic features of included participant.

**Variable** E	Overall *n*=134
**ADNC^a^**	83^b^ (62.8%)
** Braak NFT stages 0-II**	25 (18.9%)
** Braak NFT stages III-IV**	21 (15.9%)
** Braak NFT stages V-VI**	35 (26.52%)
**PART^a^**	49 (37.1%)
** Braak NFT stages 0-II**	44 (33.3%)
** Braak NFT stages III-IV**	5 (3.8%)
**Thal Aβ phases^a^**	
** Phase 0**	17 (12.9%)
** Phase 1**	32 (24.2%)
** Phase 2**	5 (3.9%)
** Phase 3**	27 (20.5%)
** Phase 4**	10 (7.6%)
** Phase 5**	41 (31.1%)
**LATE-NC stages**	
** Stage 0**	62 (46.3%)
** Stage 1**	27 (20.1%)
** Stage 2**	33 (24.6%)
** Stage 3**	12 (9.0%)
**LC pTDP-43 pathology**	
** None**	106 (79.1%)
** RG(s) only**	17 (12.7%)
** RG(s) + other**	10 (7.5%)
** Other only**	1 (0.7%)

Abbreviations: ADNC, Alzheimer disease neuropathologic change; LC, locus coeruleus; NFT, neurofibrillary tangle; RG, round granule.

a *n* = 132 participants with Braak NFT staging and/or Thal Aβ phase.

b *n* = 2 participants had missing Braak NFT staging but Thal Aβ phase >1.

### Findings related to pTDP-43 and pTau pathologies

Among the included participants, 62.8% (*n* = 83/132) had any ADNC pathology, and 53.7% (*n* = 72) had LATE-NC stage >0 (Table 2). On reassessment of MTL regions with LATE-NC stage 0 and LC pTDP-43 pathology, one case with subtle amygdala pTDP-43 pathology was reclassified to LATE-NC stage 1.

**Figure 2:**
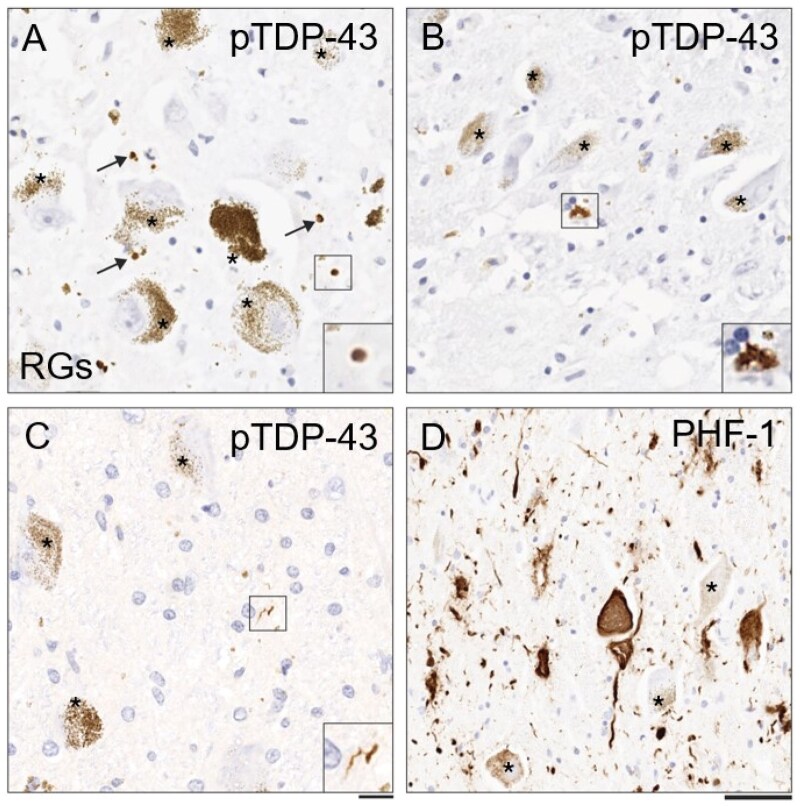
Photomicrographs of locus coeruleus (LC) with immunohistochemical staining of pathology (brown), counterstained with hematoxylin (blue). (A-C) LC pTDP-43 pathology. Note that LC neurons also contained pigmented neuromelanin granules, denoted with asterisks (*). Arrows and inset in panel A indicate individual pTDP-43+ round granules (RGs). Examples of other LC pTDP-43 pathology are depicted in panels B and C. Some lesions appeared as NCIs (B) or dystrophic neurites (C). A photomicrograph of immunoreactive phosphorylated tau (pTau, stained with antibody PHF-1) demonstrating that pTau pathology was oftentimes more severe in the LC (D). Scale bar is 50 μm, with the inset scale bar at 10 μm.

**Figure 3:**
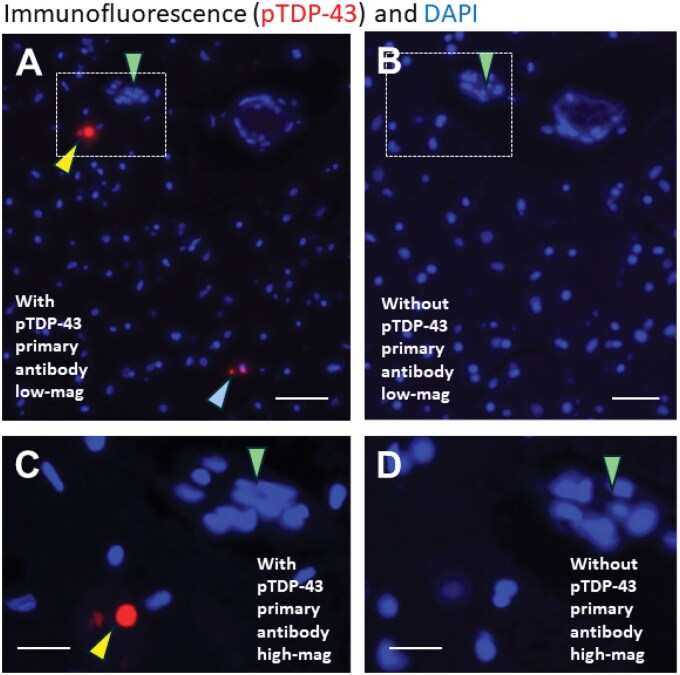
pTDP-43+ round granules (RGs) in the locus coeruleus (LC) were only seen in sections where the pTDP-43 primary antibody was included. This experiment was necessary to show the specificity of RG staining given the potential for conflation with neuromelanin. Shown here are photomicrographs depicting serial sections of an LC stained for pTDP-43 (red fluorophore-conjugated secondary antibody), with (A, C) or without (B, D) the primary pTDP-43 antibody included. Sections were counterstained for DAPI which labels viable nuclei. Panels A and B are low-magnification photomicrographs, C (corresponding with A) and D (corresponding to B) are high-magnification. The same cell group (a small blood vessel) is indicated in all 4 panels with a green arrowhead. pTDP-43 pathology in panel A is indicated with a yellow arrowhead and a pale-blue arrowhead. In panel C, the RG depicted with the yellow arrowhead is shown at higher magnification. In the serial section (B, D) for which the staining was performed exactly the same but pTDP-43 primary antibody was not included, no pTDP-43+ RG was seen. Scale bar = 150 μm (A, B) and 40 μm (C, D).

Overall, 20.9% (*n* = 28) of participants had pTDP-43 pathology in the LC (Table 2). When present, LC pTDP-43 proteinopathy was typically sparse and showed a limited range of histopathologic appearances. Immunohistochemically stained LC pTDP-43 lesions appeared most commonly as roughly spherical deposits with a granular appearance, measuring approximately 3-12 µm in diameter, which were interpreted as extracellular upon histopathological examination. These structures were classified and counted as “RGs” (Figure 2A). Of the *n* = 28 participants with pTDP-43 pathology, *n* = 17 (12.7% of total participants and 60.7% of LC pTDP-43+ cases) had only RG lesions. In other words, although they were pTDP-43 pathology-positive, most RG-harboring LCs did not have any apparent NCIs or DNs, as would be seen more commonly in cortical pTDP-43 pathology. Immunofluorescence staining further indicated that RGs were extracellular structures and were distinct from neuromelanin granules and clusters. Round granules did not appear to colocalize with cells harboring viable nuclei as visualized by DAPI (Figure 3A and C). Moreover, RGs were not seen in preparations performed without the primary anti-pTDP-43 antibody (Figure 3B and D). We also stained pTDP-43+ LCs with antibodies raised against nonphosphorylated TDP-43 pathology; this confirmed the presence of LC pTDP-43 pathology including RGs (Figure S1). In a limited sample, we additionally used IF staining to colocalize pTDP-43 and GFAP, a marker of reactive astrocytes. GFAP generally did not colocalize with pTDP-43 but tended to surround some pTDP-43+ RGs (Figure S2). Other pTDP-43-immunostained structures (pTDP-43+ NCIs, skeins, or neurites), more similar to what is seen in other common TDP-opathies, were apparent in the LC of 11 participants (Figure 2B and C).

**Figure 4:**
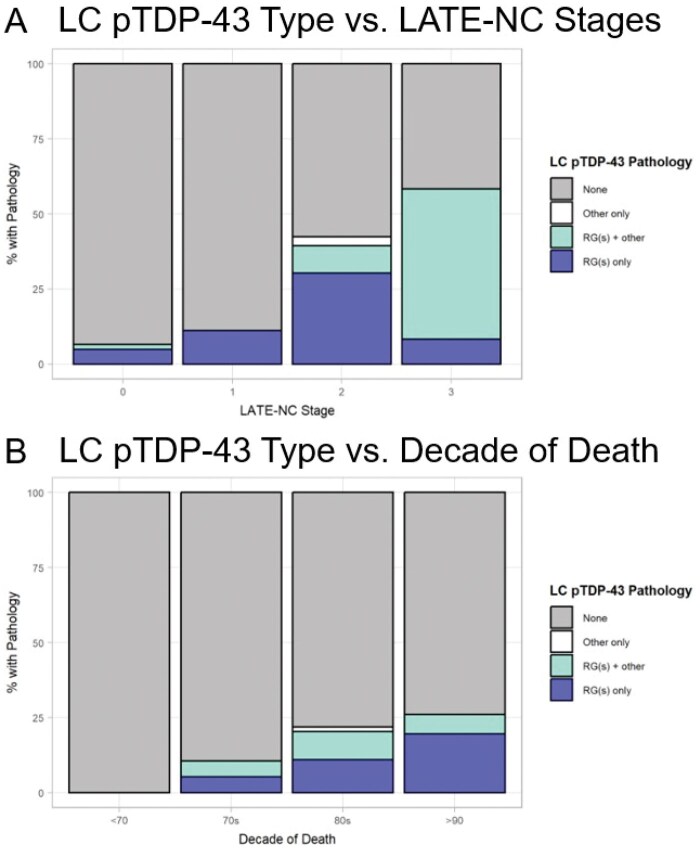
Distribution of pTDP-43 pathologic presence and subtypes, stratified by LATE-NC stages (A) and decade of death (B). Each column represents the percent of the total sample within each group (either LATE-NC stage or decade of death). The frequency of LC pTDP-43 pathology in the LC increased with LATE-NC stages and with ages of death.

The odds of having LC pTCP-43 pathology present significantly increased with LATE-NC stages >1 (*P* < .001; Table 3). Although the percent of individuals with pTDP-43 pathology in their LC increased with LATE-NC stages, the frequency of brains with only RG lesions declined in LATE-NC stage 3, in which cases pTDP-43 pathology tended to include NCIs and DN, often in addition to RGs (Figure 4A). Despite the statistically significant association between LC pTDP-43 presence and LATE-NC stages, the 2 types of pTDP-43 pathologies were not perfectly aligned: 6.5% (*n* = 4/62) of individuals with LATE-NC stage 0 had detectable LC pTDP-43 pathology, whereas only 58.3% (*n* = 7/12) of individuals with LATE-NC stage 3 had LC pTDP-43 pathology (Table 4). We reassessed the 4 cases with LATE-NC stage 0 (immunostained serial sections) and confirmed the presence of pTDP-43+RGs in those cases (data not shown).

**Table 3:** LC pTDP-43 pathology (presence and counted number of lesions per case) as they relate to age, sex, LATE-NC stages, and Braak NFT stages.

Variable	Logit model		Count model	
	Odds ratio (95% CI)	*P*-value	Relative risk (95% CI)	*P*-value
**Age at death**	1.1 (1.0-1.2)	**.025**	1.0 (0.9-1.1)	.996
**Sex**	1.5 (0.6-4.1)	.411	0.8 (0.4-1.7)	.556
**LATE-NC stage > 1**	8.5 (3.0-24.3)	**<.001**	1.8 (0.7-4.4)	.226
**Braak NFT stage > 4**	1.6 (0.6-4.7)	.366	0.8 (0.4-1.6)	.493

Abbreviations: LATE-NC, limbic predominant age-related TDP-43 encephalopathy neuropathologic change; LC, locus coeruleus; NFT, neurofibrillary tangle.

**Table 4.** Percent with any LC pTDP-43 pathology, stratified by LATE-NC stages.

Variable	**Overall** *n*=134	**LATE-NC stage**			
		**0**	**1**	**2**	**3**
		***n*=62**	***n*=27**	***n*=33**	***n*=12**
**Participants with LC pTDP-43 pathology^a^**	28 (20.9%)	4 (6.5%)	3 (11.1%)	14 (42.4%)	7 (58.3%)
** RG(s) only**	17 (12.7%)	3 (4.8%)	3 (11.1%)	10 (30.3%)	1 (8.3%)
** RG(s) + other**	10 (7.5%)	1 (1.6%)	0 (0.0%)	3 (9.1%)	6 (50.0%)
** Other only**	1 (0.7%)	0 (0.0%)	0 (0.0%)	1 (3.0%)	0 (0.0%)
**Participants with no LC pTDP-43 pathology^a^**	106 (79.1%)	58 (93.5%)	24 (88.9%)	19 (57.6%)	5 (41.7%)

Abbreviations: LATE-NC, limbic predominant age-related TDP-43 encephalopathy neuropathologic change; LC, locus coeruleus; RG, round granule.

a *n* (%).

Locus coeruleus pTDP-43 pathology was positively correlated with aging; a lower percentage of individuals who died in their seventh decade had detectable LC pTDP-43 pathology (10.5%) in comparison to those who died beyond the age of 90 years (26.1%) (Figure 4B). Accordingly, after adjusting for potential confounding variables, a statistically significant association was observed between the presence of LC pTDP-43 pathology and age at death (*P* = .025; Table 3). By contrast, no association was found between LC pTDP-43 pathology and sex (Table 3).

As expected, pTau pathology was commonly present in the LC. Assessed independently, both LATE-NC stages (*P* = .004) and Braak NFT stages (*P* < .001) were positively correlated with LC pTau pathology (Table S4). To further understand the relationship between LC pTDP-43, LC pTau, and cortical pTau pathologies, digitally quantified counts of MTL and neocortical pTau pathology were incorporated into the study. These metrics enabled separate analyses of correlations between the cortical pTau pathology in PART (where pTau is largely confined to the MTL) and ADNC (where pTau often extends into the neocortex) with regard to pathology in the LC.

**Figure 5:**
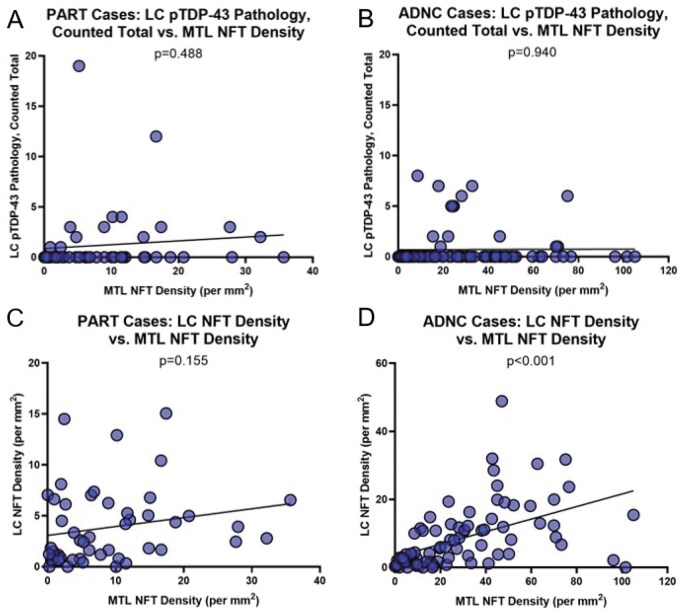
Locus coeruleus and MTL pathological burdens in the context of PART (A, C) and ADNC (B, D). Locus coeruleus pTDP-43 pathology was not associated with MTL NFT density in PART or ADNC (A-B). Locus coeruleus NFT density was not associated with MTL NFT density in PART (C), but was associated with MTL NFT pathology in ADNC (D).

Among participants with PART, LC pathologic burden (pTau and pTDP-43 lesions) did not significantly correlate with MTL pTau pathologic burden (*P* = .488) (Figure 5A), nor did the number of LC pTDP-43 lesions correlate with LC NFT density (*P* = .367) (Figure S3A). Likewise, in brains with ADNC (ie, Thal Aβ phase >1), LC pTDP-43 pathology counts were not associated with NFT densities in the MTL (*P* = .940), neocortex (*P* = .915), or LC (*P* = .078) (Figures 5B and S3B, C). Further, LC pTDP-43 pathology, both in terms of presence (*P* = .366) and severity (*P* = .493), was not significantly associated with high ADNC (Braak NFT stages V and VI; Table 3). In contrast to pTDP-43 pathology, within ADNC cases, LC pTau (as operationalized by NFT density) was strongly correlated with NFT density in the MTL (*P* < .001) (Figure 5D) and in the neocortex (*P* < .001) (Figure S3D).

**Figure 6:**
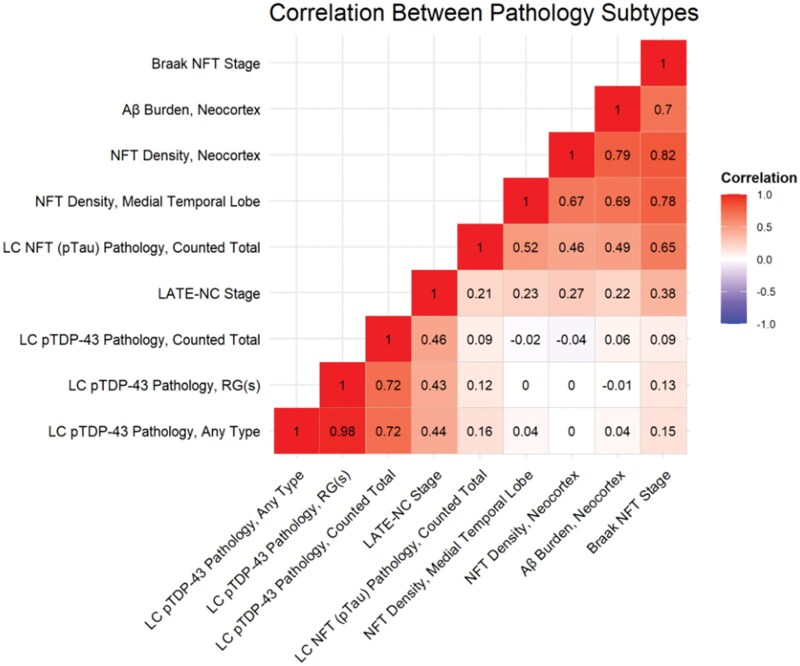
Heatmap of correlations between pathology subtypes. Locus coeruleus and cortical pathological measures were compared to assess their degree of correlation. Quantitative cortical data included Aβ burden and NFT density, with Braak NFT staging and LATE-NC staging included as additional measures of cortical pTau and pTDP-43 pathologies. Correlations between variables were calculated with Pearson, polyserial, or polychoric correlation analyses depending on their variable type.

When compared using various correlative models, accounting for both continuous and ordinal data types, ADNC associated variables (LC, MTL, and neocortical NFT density; neocortical Aβ burden; and Braak NFT staging) and pTDP-43 pathology measurements (LC pTDP-43 variables and LATE-NC staging) generally demonstrated stronger association within their respective categories (pTDP-43 or ADNC). However, it was notable that LATE-NC stages demonstrated a stronger relationship with ADNC-associated pathology than LC pTDP-43 pathology did (Figure 6).

### Associations between pathologic readouts and antemortem clinical symptoms

**Figure 7:**
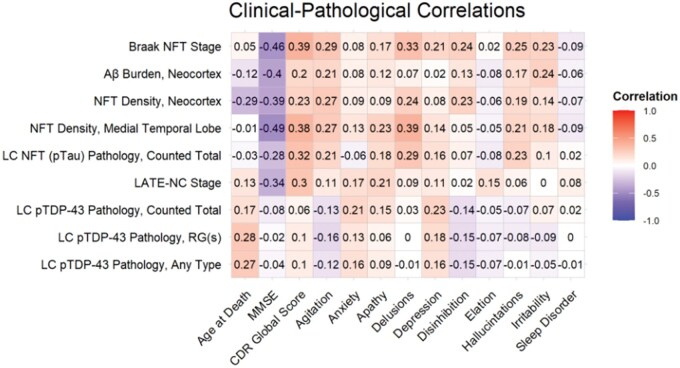
Heatmap of clinical-pathological correlations. Age at death, cognition measures, and BPSD were compared to LC pathology in addition to previously assessed cortical pathologic (ADNC and LATE-NC) data. Correlations between variables were calculated with Pearson, polyserial, or polychoric correlation analyses depending on their variable type. Abbreviations: ADNC, Alzheimer disease neuropathologic change; BPSD, behavioral and psychiatric symptoms of dementia; LATE-NC, limbic predominant age-related TDP-43 encephalopathy neuropathologic change.

Locus coeruleus pTDP-43 and pTau pathologies were tested for their associations with BPSD among the 124 participants with available data. A regression-based hurdle model was employed to assess the impact of LC pTDP-43 pathology (presence and count) on caregiver reported BPSD, whilst accounting for the weight of other covariates. The only significant relationship was a positive association between the number of LC pTDP-43 lesions (the severity of pathology) and NPI-Q depression (*P* = .028) (Table 5). The presence or absence of LC pTDP-43 pathology, that is, testing as a dichotomous variable, did not show any significant correlations with BPSD (Table S5). Locus coeruleus pTDP-43 pathologic measures tended toward stronger positive correlation with depression than with other BPSD, even when using a less sophisticated statistical test (Figure 7). In contrast, increased NFT density within the LC was significantly associated with apathy (*P* = .043), delusions (*P* = .031), and depression (*P* = .043), when assessed using a Wilcoxon Rank Sum Test, which did not account for covariates (Table S6). Since at least one NFT was digitally detected in the LC of most individuals (94.0%; *n* = 126/134), it was not necessary to run a presence vs absence analysis of pTau pathology.

**Table 5:** LC pTDP-43 pathology counts, and association with behavioral and psychiatric symptoms of dementia (BPSD).

	**Count model** ^a^	
Variable	Relative risk (95% CI)	*P*-value
**Agitation**	0.5 (0.1-1.6)	.244
**Anxiety**	1.9 (0.8-4.8)	.148
**Apathy**	1.8 (0.7-4.4)	.232
**Delusions**	1.6 (0.3-7.8)	.533
**Depression**	2.4 (1.1-5.1)	**.028**
**Hallucinations**	0.2 (0.0-1.6)	.149
**Sleep disorders**	2.2 (0.5-9.1)	.260
**Disinhibition^b^**	–	–
**Elation^b^**	–	–

Abbreviations: LATE-NC, limbic predominant age-related TDP-43 encephalopathy neuropathologic change; LC, locus coeruleus; NFT, neurofibrillary tangle.

a Model adjusted for age at death, sex, LATE-NC stage >1, and Braak NFT stage >IV.

b Insufficient number of participants with scored disinhibition or elation and LC pTCP-43 pathology counts for hurdle model (*n* < 3).

No negative association was observed between LC pTDP-43 pathology and global cognitive impairment. When stratifying by LATE-NC stage, there was a trend toward higher MMSE scores in cases with LC pTDP-43 present compared to those without LC pTDP-43 pathology (Table 6). This association was not investigated statistically. The observed trend of increased MMSE scores with LC pTDP-43 pathology was in contrast to the robust inverse association between MMSE scores and other pathological subtypes (ADNC and LATE-NC; Figure 7).

**Table 6:** Final average (SD) MMSE scores stratified by LATE-NC stage and LC pTDP-43 presence.

**Variable**	**LC pTDP-43 pathology**	**LATE-NC stage**			
**Variable**		0 (*n*=57)	1 (*n*=27)	2 (*n*=31)	3 (*n*=12)
**MMSE scores^a^**	Present	28.0 (1.7)	26.7 (4.2)	21.7 (7.1)	21.0 (4.4)
	Absent	25.2 (6.5)	23.2 (7.3)	19.5 (7.9)	18.2 (11.7)

Abbreviations: LATE-NC, limbic predominant age-related TDP-43 encephalopathy neuropathologic change; LC, locus coeruleus; MMSE, Mini Mental State Exam.

a Mean (SD).

## Discussion

In this study, we describe LC pTDP-43 pathology in aged human brains from the UK-ADRC community-based autopsy cohort. In this convenience sample, 20.9% of brains had pTDP-43 pathology in their LC. This pTDP-43 pathology was relatively sparse and tended to appear as RG lesions. Locus coeruleus pTDP-43 pathology demonstrated an imperfect correlation with LATE-NC stages: several participants with LATE-NC stage 0 had LC pTDP-43 pathology present, whereas some cases with LATE-NC stage 3 lacked any detected LC pTDP-43 pathology. Increasing quantitative LC pTDP-43 burden demonstrated a nominally significant positive association with caregiver rated symptoms of depression, based on lesion counts rather than the binary presence or absence of LC pTDP-43 pathology.

The LC has been a hot spot for research in neurodegenerative diseases. For example, LC pTau pathology has been evaluated in previous autopsy-based studies. These studies have shown that pTau pathology accumulates in the LC of most adults and that the severity of LC pTau pathology has been linked to age-related cognitive decline.^26^^,^^63^ Additionally, previous studies showed that the severity of LC pTau pathology was associated with Braak NFT staging,^19^^,^^21^^,^^64^ which was replicated in our current findings. Notably, another study observed a plateau in LC pTau NFTs at higher stages of ADNC severity and identified a negative correlation between LC neuron count and Braak NFT staging, which indicates a more complex role between pTau and LC neuron integrity.^20^ That being said, other misfolded proteins, such as Aβ and α-synuclein (α-syn), have also been investigated in the LC. Beardmore et al. found a significant association between LC Aβ load and Braak NFT stages V and VI compared to lower Braak NFT stages.^21^ A recent investigation of α-syn pathology in the LC found that most individuals with ADNC or LBD had LC α-syn immunoreactivity, although this proportion differed when using different anti-α-syn antibodies.^65^

As far as we are aware, only one prior published study has systematically evaluated LC pTDP-43 pathology.^56^ That study primarily focused on LC degeneration across various subtypes of FTLD among participants recruited from a memory disorder clinic. Their findings indicated a greater severity of LC pTDP-43 pathology (when present) than we see in our community-based cohort, in which LATE-NC, rather than FTLD-TDP, is more predominant. Compared to brains with LATE-NC, FTLD-TDP brains have a more widespread distribution of severe pTDP-43 proteinopathy.^66–69^ For example, in the frontal cortex, pTDP-43 pathology in FTLD-TDP types A and B is typically 1 or 2 orders of magnitude more dense than that seen in LATE-NC stage 3.^68^^,^^69^ Notably, the study of Ohm et al. did not find an association between LC pTDP-43 pathology and LC neuronal degeneration.^56^

The current study of LC pTDP-43 pathology produced several remarkable findings. These included a general lack of correlations between LC pTDP-43 pathology and clinical symptoms, and a positive correlative relationship between LC NFT density and LATE-NC stages. In regard to LC pTDP-43 pathology itself, additional observations included the presence of a distinct pTDP-43 morphological lesion (RGs), the relative sparseness of the pathology, and a significant, though imperfect, correlation with increasing LATE-NC stages.

Most LCs with pTDP-43 pathology contained at least one RG; only one individual had LC pTDP-43 pathology without an observed RG (Table 4). Round granules are small, roughly spherical appearing pTDP-43+ lesions (Figure 2A), and did not appear to colocalize with viable nuclei (Figure 3A and C) but in some cases appeared to be embraced by GFAP-immunoreactive astrocyte processes (Figure S2). Subtypes of pTDP-43 pathology appeared to change with respect to LATE-NC stage, but not with respect to age of death (Figure 4). Most individuals with LC pTDP-43 lesions in LATE-NC stages 0-2 only presented with RG lesions. However, among the included cases of LATE-NC stage 3 (*n* = 12), RG pathology was frequently accompanied by additional pTDP-43 subtypes, such as NCIs, suggesting a shift toward a more complex pathological profile in the most severe stage of disease. This apparent stage-specific pattern of LC pTDP-43 lesion development suggests a relationship between pTDP-43 pathological subtypes in the LC with cortical LATE-NC staging, possibly related to pathological spread.

Contrary to our expectations, the presence of pTDP-43 pathology in the LC did not perfectly align with LATE-NC stages. While individuals with LATE-NC stage 2 or 3 were significantly more likely to exhibit at least one pTDP-43 lesion (*P* < .001), and the proportion of individuals in each LATE-NC stage with LC pTDP-43 lesions increased across stages (Figure 4A), only 58.3% of those with LATE-NC stage 3 had detectable pTDP-43 pathology in the LC. This percentage was similar to the proportion of FTLD-TDP individuals with LC pTDP-43 immunoreactivity in the study of Ohm et al.,^56^ but exceeds the frequency of subcortical pTDP-43 pathology in LATE-NC as reported by Josephs et al.^31^^,^^32^^,^^36^ Notably, 6.5% of individuals with LATE-NC stage 0 exhibited LC pTDP-43. Despite the fact that these cases had no detectable pTDP-43 pathology in regions routinely screened during diagnostic procedures (MTL and MFG), this compact cell group (with a much smaller surface area than stained MTL/MFG regions) did contain pTDP-43-immunoreactive structures.

Although LC pTDP-43 was a major focal point of this study, LC and global pTau pathologic assessments were also of interest. Locus coeruleus NFT density was associated with Braak NFT staging (*P* < .001) and LATE-NC staging (*P* = .004). Although numerous studies have linked LC pTau pathology to Braak NFT staging,^19^^,^^21^^,^^64^ this is the first time LC pTau pathology has been linked to LATE-NC staging. The findings outlined here further support the hypothesis that increased pTau aggregation is associated with the severity of LATE-NC and/or cortical pTDP-43 pathology.^35–37^ Whereas cortical pTDP-43 proteinopathy (as operationalized by LATE-NC staging) was associated with LC NFT density, the density of NFTs in the LC did not significantly correlate with LC pTDP-43 counts (Figure S1). Further, severe ADNC (Braak NFT stages V or VI) was not significantly associated with the presence or severity of LC pTDP-43 pathology (Table 3). These observations, along with the distinct morphological features of RGs as a pathological lesion subtypes, suggest mechanism(s) of LC pTDP-43 pathology that are at least somewhat distinct from those involved with cortical pTDP-43 pathology.

We also observed different correlative patterns of LC pTau aggregation in comparing between cases with PART and ADNC. In PART, LC NFT density did not significantly correlate with cortical NFT density (Figure 5). However, in brains with ADNC, LC NFT density was significantly correlated with NFT density in relevant cortical regions (Figure S1). Although pTau appears to aggregate in the LC as early as the second decade of life in most people,^18^ individuals with PART do not exhibit the same strong relationship between LC pTau and cortical pTau pathologies that those with ADNC had. Notably, LC Aβ does not tend to develop until Thal Aβ phase 5, as described by Thal et al, indicating that the LC is not affected by Aβ amyloidosis until later in ADNC progression.^51^ This is further supported by the previously observed association between LC Aβ load and high Braak NFT stages, again indicating that Aβ impacts the LC relatively late in ADNC progression.^21^ Since Aβ amyloidosis is the differentiating criterion between PART and ADNC, this supports the hypothesis that there is a possible role for cortical Aβ in LC NFT development. Further, this distinction between LC pTau in PART and ADNC is yet more indication that these 2 conditions are separable pathological entities.

Another topic addressed by the current study was the association between LC pTDP-43 pathology and clinical features of dementia, including BPSD. The LC and its dysfunction are known to be associated with psychiatric disorders, consistent with the integral role in brain norepinephrine innervation of this cell group.^2^^,^^5^^,^^7^^,^^8^ Behavioral and psychiatric symptoms of dementia have been associated with reduced LC integrity detected via neuroimaging^27^ and with subcortical pTau pathology in the LC, even in low Braak NFT stages.^28^ In the current study, LC pTDP-43 pathologic counts, when analyzed with a model that accounted for potential confounding variables, were positively associated with depression (*P* = .028) (Table 5). Yet in the same study sample, individuals with LC pTDP-43 pathology tended to have higher MMSE scores. Thus, LC pTDP-43 may have a relatively benign association with global cognition in contrast to its possible link with depressive symptoms. These findings may suggest a distinct role of LC pTDP-43 pathology in neuropsychiatric dysfunction. Given the modestly sized convenience sample in the study, the results need to be further tested in future studies, however.

Lewy body pathology (LBP) also has been associated with clinically defined neuropsychiatric conditions such as depression and anxiety^70–72^; and LBP-associated degeneration of neurons in the LC is associated with BPSD.^73^ However, only one individual in the current study had both pTDP-43 and LBD pathologies, eliminating the possibility of statistical evaluations when these 2 pathologies were comorbid. As a result, the potential interaction between these 2 types of LC pathologies remains unclear.

This study has limitations. The sample included participants who were mostly highly educated and White. The present study also involved a broad range of correlation analyses related to both pathological and clinical variables. However, the sample size of the study constrained the statistical power. Future work will be required in larger, more diverse and generalizable samples. For operationalization of BPSD, we used NPI-Q ratings based on assessments rendered by a study partner, whereas clinical diagnoses may have yielded different results. Yet the relationships observed in the current study help set the stage for more focused future investigations. In regard to the neuropathologic evaluations, there were technical limitations and pitfalls, particularly related to histologic analyses in LC sections. Although only pons sections with LC tissue were included and efforts were made to select tissue sections with consistent anatomical landmarks (rostral pons), there was some variability in the level of sectioning. This is an important caveat given prior evidence of differential vulnerability to pathology along the length of the LC in both ADNC and LBP.^9^^,^^11^^,^^13^^,^^57^^,^^74–76^ In future studies, we would aim to deploy a study design that more systematically dissects, sections, and evaluates the full rostral-caudal extent to the LC.

## Conclusion

This study represents the first focused examination of pTDP-43 pathology in the LC in the context of the aging human brain and prevalent dementia-related copathologies such as LATE-NC and ADNC. Even in brains where LC pTDP-43 pathology was detected using IHC, the detected pathology was relatively sparse. Locus coeruleus pTDP-43 pathology exhibited a statistically significant association with LATE-NC stages and age of death. There was relatively weak evidence of association between LC pTDP-43 pathology and ADNC-related changes, whereas LC pTau pathology was linked to LATE-NC staging. These results highlight potential spatial nuances in pTau and pTDP-43 pathologic mechanisms. Further, there was an association between LC pTDP-43 pathology counts and depressive symptoms but, overall, the correlations between LC pTDP-43 pathology and clinical parameters were weak. Based on our findings, we do not see compelling evidence in favor of LC pTDP-43 pathology being incorporated into a neuropathology-based staging or classification system of aging-related TDP-43 proteinopathy or LATE-NC.

## Supplementary Material

nlag035_Supplementary_Data

## Data Availability

All data used in this article will be made available to the research community in a reasonable timeframe.
