# Supplementary material for: Locus coeruleus TDP-43 pathology in a community-based cohort: Clinical and pathological correlates
Source: J Neuropathol Exp Neurol. 2026 Apr 20;85(8):842–56. doi: 10.1093/jnen/nlag035 (PMC13440673; doi:10.1093/jnen/nlag035)
Supplement: Supplementary file 1 [file nlag035_supplementary_data.zip › Nelson Supplementary Tables.docx]

**Supplemental Table 1. Participant BPSD frequencies**

| **Variable** | n | **Overall^1^** |
| --- | --- | --- |
| Agitation | 124 | 37 (29.8%) |
| Anxiety | 124 | 40 (32.3%) |
| Apathy | 124 | 43 (34.7%) |
| Delusions | 122 | 11 (9.0%) |
| Depression | 124 | 42 (33.9%) |
| Disinhibition | 124 | 22 (17.7%) |
| Elation | 124 | 2 (1.6%) |
| Hallucinations | 123 | 12 (9.8%) |
| Irritability | 124 | 32 (25.8%) |
| Sleep disorder | 124 | 22 (17.7%) |
| ^1^n (%) | | |

**Supplemental Table 2. NFT density within the neocortex and medial temporal lobe (MTL)**

| **Region** | **Subregion** | **NFT Density/mm^2^** |
| --- | --- | --- |
| **Neocortical^1^** (n=134) |  |  |
|  | Middle frontal gyrus | 3.4 (10.6) |
|  | Inferior parietal lobule | 3.4 (7.7) |
|  | Temporal (SMTG) neocortex | 7.4 (14.6) |
|  | Occipital (striate) neocortex | 2.2 (6.9) |
|  | **Neocortex average** | **4.1 (8.4)** |
| **MTL^1^** (n=133) |  |  |
|  | Amygdala | 16.8 (22.7) |
|  | Entorhinal cortex | 22.2 (20.0) |
|  | CA1 | 29.5 (37.4) |
|  | Subiculum | 19.9 (24.3) |
|  | **MTL average** | **21.8 (22.7)** |

^1^Mean (SD)

**Supplemental Table 3. Neuropathologic features of included subjects as stratified by sampling group.**

| **Variable** | **CERAD 0** | **2022-2024** | **LATE-NC Stage 3** | **Overall** | |
| --- | --- | --- | --- | --- | --- |
|  | **N=69** | **N=56** | **N=9** | **N = 134** | |
| **ADNC*** | 35** (50.7%) | 43** (76.8%) | 5 (55.6%) | 83** (62.8%) | |
| Braak NFT 0-II | 21 (30.9%) | 4 (7.3%) | 0 (0.0%) | 25 (18.9%) | |
| Braak NFT III-IV | 13 (19.1%) | 6 (10.9%) | 2 (22.2%) | 21 (15.9%) | |
| Braak NFT V-VI | 0 (0.0%) | 32 (58.2%) | 3 (33.3%) | 35 (26.52%) | |
| **PART*** | 34 (49.3%) | 13 (23.2%) | 2 (22.2%) | 49 (37.1%) | |
| Braak NFT 0-II | 33 (48.5%) | 9 (16.4%) | 2 (22.2%) | 44 (33.3%) | |
| Braak NFT III-IV | 1 (1.5%) | 4 (7.3%) | 0 (0.0%) | 5 (3.8%) | |
| **Thal Aβ phases*** |  |  |  |  | |
| Stage 0 | 13 (18.8%) | 4 (7.1%) | 0 (0.0%)*** | 17 (12.9%) | |
| Stage 1 | 21 (30.4%) | 9 (16.1%) | 2 (28.6%)*** | 32 (24.2%) | |
| Stage 2 | 5 (7.2%) | 0 (0.0%) | 0 (0.0%)*** | 5 (3.9%) | |
| Stage 3 | 24 (34.8%) | 3 (5.4%) | 0 (0.0%)*** | 27 (20.5%) | |
| Stage 4 | 2 (2.9%) | 5 (8.9%) | 3 (42.9%)*** | 10 (7.6%) | |
| Stage 5 | 4 (5.8%) | 35 (62.5%) | 2 (28.6%)*** | 41 (31.1%) | |
| **LATE-NC** |  |  |  |  | |
| Stage 0 | 39 (56.5%) | 23 (41.1%) | 0 (0.0%) | 62 (46.3%) | |
| Stage 1 | 20 (29.0%) | 7 (12.5%) | 0 (0.0%) | 27 (20.1%) | |
| Stage 2 | 9 (13.0%) | 24 (42.9%) | 0 (0.0%) | 33 (24.6%) | |
| Stage 3 | 1 (1.4%) | 2 (3.6%) | 9 (100.0%) | 12 (9.0%) | |
| **LC pTDP-43 pathology** |  |  |  |  | |
| None | 61 (88.4%) | 41 (73.2%) | 4 (44.4%) | 106 (79.1%) | |
| RG(s) only | 6 (8.7%) | 10 (17.9%) | 1 (11.1%) | 17 (12.7%) | |
| RG(s) + other | 2 (2.9%) | 4 (7.1%) | 4 (44.4%) | 10 (7.5%) | |
| Other only | 0 (0.0%) | 1 (1.8%) | 0 (0.0%) | 1 (0.7%) | |
| *n=132 participants with Braak NFT staging AND/OR Thal Aβ phase | | | | |  |
| **n=2 participants had missing Braak NFT staging but had Thal Aβ phase > 1; one was in the 2022-2024 group and one was in the CERAD 0 group. ADNC/PART percentages are calculated with N=69 and N=56 respectively; stratified Braak NFT staging percentages are calculated with N=68 and N=55  ***Out of n=7 LATE-NC stage 3 group individuals with recorded Thal Aβ Phase | | | | |  |

**Supplemental Table 4. Associations between LC-NFT density with LATE-NC stage and Braak NFT stage**

|  | **LATE-NC Stage** | | | |  |
| --- | --- | --- | --- | --- | --- |
|  | **0**    **N = 62** | **1**    **N = 27** | **2**    **N = 33** | **3**    **N = 12** | **p-value** |
| LC NFT Density (NFT/mm^2)^1^ | 2.2 (0.8, 7.0) | 2.8 (1.7, 7.4) | 8.7 (3.3, 15.4) | 6.0 (2.7, 12.3) | **0.004^2^** |
| ^1^Median (Q1, Q3) | | | | | |
| ^2^Kruskal-Wallis Test | | | | | |

|  | **Braak NFT Stage** | | | | | | |  |
| --- | --- | --- | --- | --- | --- | --- | --- | --- |
|  | **0**    **N = 4** | **I**    **N = 28** | **II**    **N = 37** | **III**    **N = 21** | **IV**   **N = 6** | **V**    **N = 30** | **VI**    **N = 6** | **p-value** |
| LC NFT Density (NFT/mm^2)^1^ | 0.5 (0.2, 0.9) | 1.6 (0.8, 3.2) | 2.5 (0.8, 5.0) | 3.9 (1.9, 5.9) | 4.7 (1.7, 8.0) | 12.5 (10.7, 20.0) | 13.6 (8.9, 18.1) | **<0.001^2^** |
| ^1^Median (Q1, Q3) | | | | | | | | |
| ^2^Kruskal-Wallis Test | | | | | | | | |

**Supplemental Table 5. LC pTDP-43 pathology presence, stratified by BPSD**

|  | **Logit Model*** | |
| --- | --- | --- |
| **Variable** | **Odds Ratio (95% CI)** | **p-value** |
| Agitation | 0.3 (0.1, 1.3) | 0.108 |
| Anxiety | 2.1 (0.7, 6.0) | 0.182 |
| Apathy | 1.2 (0.4, 3.5) | 0.737 |
| Delusions | 0.5 (0.1, 3.2) | 0.439 |
| Depression | 1.5 (0.5, 4.3) | 0.429 |
| Hallucinations | 0.5 (0.1, 3.2) | 0.476 |
| Sleep disorder | 0.5 (0.1, 1.9) | 0.279 |
| Disinhibition** | -- | -- |
| Elation** | -- | -- |

*-Models are adjusted for age at death, sex, LATE-NC Stage > 1, and Braak NFT Stage > IV.

**-Insufficient number of participants with elation or disinhibition and LC pTCP-43 pathology counts for hurdle model (n<3)

**Supplemental Table 6. LC NFT density, stratified by BPSD**

|  | **BPSD Absent** | | **B{SD Present** | |  |
| --- | --- | --- | --- | --- | --- |
| **Variable** | **n** | **Median (IQR)** | **n** | **Median (IQR)** | **p-value^1^** |
| Agitation | 87 | 2.9 (1.2, 7.7) | 37 | 5.3 (2.1, 12.9) | 0.074 |
| Anxiety | 84 | 3.9 (1.2, 9.2) | 40 | 3.9 (1.8, 10.8) | 0.528 |
| Apathy | 81 | 2.8 (1.1, 8.1) | 43 | 5.0 (2.3, 13.6) | **0.043** |
| Delusions | 111 | 2.9 (1.2, 8.2) | 11 | 6.7 (3.6, 14.9) | **0.031** |
| Depression | 82 | 3.6 (1.1, 7.8) | 42 | 5.0 (1.9, 13.8) | **0.043** |
| Disinhibition | 102 | 3.1 (1.2, 8.6) | 22 | 5.3 (2.4, 11.6) | 0.124 |
| Elation | 122 | 3.9 (1.4, 10.3) | 2 | 1.3 (1.1, 1.5) | 0.230 |
| Hallucinations | 111 | 3.8 (1.2, 8.5) | 12 | 6.6 (3.2, 18.4) | 0.056 |
| Irritability | 92 | 3.9 (1.2, 8.8) | 32 | 3.8 (1.8, 10.9) | 0.646 |
| Sleep disorder | 102 | 4.0 (1.4, 10.3) | 22 | 2.5 (1.2, 8.1) | 0.697 |
| ^1^Wilcoxon Rank Sum Test | | | | | |
