# Supplementary material for: Locus coeruleus TDP-43 pathology in a community-based cohort: Clinical and pathological correlates
Source: J Neuropathol Exp Neurol. 2026 Apr 20;85(8):842–56. doi: 10.1093/jnen/nlag035 (PMC13440673; doi:10.1093/jnen/nlag035)
Supplement: Supplementary file 1 [file nlag035_supplementary_data.zip › Nelson Supplementary Figures.docx]

**Supplemental Figures**

pTDP-43

Non-phos- TDP-43

**C**

**D**

**Supplemental Figure 1. Photomicrographs showing the appearance of different chromagen (A: 3,3’-diaminobenzidine, or DAB; and, B: NovaRED) and antibodies that recognize either phosphorylated (C) or non-phosphorylated TDP-43 epitopes (C,D) in serial sections of pons tissue.** DAB (A) and Nova Red (B) chromagens were compared on pTDP-43 IHC stained serial sections of LC. Additionally, we compared antibodies raised against phosphorylated TDP-43 (C) and non-phosphorylated TDP-43 (D). The pTDP-43 proteinopathy included extracellular lesions with round granule morphology, whereas total TDP-43 also had widespread nuclear staining of neurons and glial cells. Black arrows point to the same neuromelanin filled neuron in A and B. Clear arrows indicate pTDP-43 proteinopathy. For A and B, scale bar is 60 µm. For C and D, scale bar is 100 µm.

DAB

NovaRED

**A**

**B**

**Supplemental Figure 2. Photomicrographs showing a locus coeruleus (LC) microscopic field from a TDP+LC that was stained for DAPI (blue); pTDP-43 immunofluorescence (IF; red); and, GFAP IF (green).** The same region is shown at relatively low power with all 3 chromagens in panel A. An area with a pTDP-43+ round granule (RG) is indicated with a yellow arrow (A) and that region is shown at higher magnification in panel B. Panels C-E show individually the same regions of the photomicrograph labeled only with DAPI (C), GFAP (D), and pTDP-43 (E). Note that one region shows co-localization with pTDP-43+RG and GFAP (yellow arrow); and another region shows pTDP-43 pathology but no GFAP colocalization (blue arrow). Note that in the RG region of colocalization the GFAP immunoreactivity is accentuated peripherally. Scale bars = 30 µm (A); 15 µm (B); and, 40 µm (C-E).

**Supplemental Figure 3. LC and neocortical pathological burdens in PART (A) and ADNC (B, C, D).** In both PART and ADNC, there was no significant association between LC neurofibrillary tangle (NFT) density and LC pTDP-43 counts (A-B). In ADNC, neocortical NFT density was not associated with LC pTDP-43 counts (C), but was associated with LC NFT density (D). Neocortical NFT density was not included for PART, since there was no Braak NFT stage V or VI case (i.e., neocortical NFTs) with PART.
